# Prevention of ulcerative colitis by Huangqin decoction: reducing the intestinal epithelial cell apoptosis rate through the IFN-γ/JAK/ETS signalling pathway

**DOI:** 10.1080/13880209.2022.2070220

**Published:** 2022-06-02

**Authors:** Xiaowei Mo, Kairui Tang, Lijing Deng, Xingyi Zhou, Xiaojuan Li, Yupei Zhang, Jing Wang

**Affiliations:** a Guangzhou Key Laboratory of Formula-Pattern of Traditional Chinese Medicine, Jinan University, Guangzhou, China; b School of Traditional Chinese Medicine, Jinan University, Guangzhou, China

**Keywords:** Traditional Chinese medicine, interferon gamma, Janus kinases

## Abstract

**Context:**

Ulcerative colitis (UC) is a chronic idiopathic inflammatory bowel disease that is closely related to inflammation and apoptosis. The traditional Chinese medicine compound preparation Huangqin decoction (HQD) has been widely used in the clinical treatment of UC, but the specific mechanism of its function is still inconclusive.

**Objective:**

To explore the pathogenesis of UC based on the IFN-γ/JAK/ETS signalling pathway, and to clarify the biological mechanism of HQD.

**Materials and methods:**

Forty 8-week-old male C57BL/6 mice were randomly divided into four groups: normal control, model, model + salazosulfapyridine group (500 mg/kg, p.o., pd) and model + HQD (9.1 g/kg, p.o., pd). Using Dextran sulphate sodium (DSS) salt (2.5%, p.o.)+high-fat diet + hot and humid environment to build a mouse model of UC. One month later, the changes of colon morphology, serum inflammatory factors, intestinal epithelial cell apoptosis and IFN-γ/JAK/ETS signalling pathway related protein changes in mice were observed.

**Results:**

Compared with the model group, HQD significantly reduced the pathological score of the model mice’s colon (2.60 ± 0.25 vs. 4.80 ± 0.37), and reduced the serum IFN-γ (200.30 ± 8.45 vs. 413.80 ± 6.97) and other inflammatory factors, and reduced intestinal epithelial cell apoptosis (24.85 ± 4.87 vs. 214.90 ± 39.21). In terms of mechanism, HQD down-regulated IFN-γ/JAK/ETS signalling pathway related proteins in colon tissue of UC model mice.

**Conclusions:**

These data indicate that HQD can improve UC by reducing intestinal inflammation and apoptosis, providing experimental evidence for the wide application of HQD in clinical practice of UC.

## Introduction

Ulcerative colitis (UC) is a chronic idiopathic inflammatory bowel disease (IBD) of the colon in which inflammation starts in the rectum and usually extends proximally to part or the entire colon in a continuous manner (Gajendran et al. 2019). In western countries, the incidence in North America is 8.8–23.14%, and the incidence in Eastern Europe is 1.7–57.9%. Currently, with the transformation of newly industrialized countries in Asia, Africa and South America to modern society or Western society, the global incidence of UC is accelerating, and it has become a global disease, with prevalence of 0.68–2.17% in Southeast Asia and 3.29–19.02% in Africa (Ng et al. 2017; Kaplan et al. 2019). Between 2002 and 2013, there were approximately 300,000 hospitalized UC patients in the United States, and 9% of them required total abdominal colectomy (Olaiya et al. 2021). In addition, due to the high recurrence rate of UC, the risk of patients developing colorectal cancer will increase significantly, which will have a serious impact on patients’ personal burden and quality of life (Zhou et al. 2019; Lichtenstein et al. 2020).

The exact cause of UC has not yet been fully elucidated, but judging from the high incidence over the past 60 years in Western countries and the current incidence of UC in developing countries, geography, age, sex, genetics, environment, gastrointestinal infection, intestinal microbiome, appendectomy and other risk factors affect the occurrence and development of UC (Gajendran et al. 2019). When discussing the pathogenesis of UC, inflammation and apoptosis seem to be the central themes. The homeostasis of colonic epithelial cells is maintained by a finely regulated balance between proliferation and apoptotic cell death (Negroni et al. 2015). The abnormally high rate of intestinal epithelial cell apoptosis in UC leads to destruction of the epithelial barrier, activation of the inflammatory response driven by the microbiota, and further progressive tissue damage (Blander 2016). The inflammatory cytokine IFN-γ is believed to be responsible for the increase in epithelial cell apoptosis observed in UC (Seidelin 2015). In classical IFN-γ signalling, binding of IFN-γ to its receptor leads to the oligomerization of the receptor subunits IFNγR1 and IFNγR2 and activation of the downstream receptor-related Janus kinases (JAK1 and JAK2), exerting numerous biological effects (Mojic et al. 2017).

Although several effective pharmaceutical preparations have been developed, including 5-aminosalicylate, corticosteroids, immunomodulators and biologics, complete cure of UC has rarely been achieved (Kato et al. 2019). In recent years, China has performed a number of multicentre randomized controlled trials of traditional Chinese medicine for the treatment of UC. The Chinese medicines in the trials have shown good efficacy, which has greatly broadened the treatment options for patients with UC (Zheng et al. 2017; Kou et al. 2020). Huangqin decoction (HQD) is a classic traditional Chinese herbal formulation consisting of four components: *Scutellaria baicalensis* Georgi (Lamiaceae), *Paeonia lactiflora* Pall (Paeoniaceae), *Glycyrrhiza uralensis* Fisch (Leguminosae) and *Ziziphus jujube* Mill (Rhamnaceae). Flavonoids and terpenoids, such as paeoniflorin, baicalein and glycyrrhizic acid, are the primary active chemical substances in HQD and have been demonstrated to have anti-inflammatory effects, repair the intestinal barrier and control intestinal microbiome effects in research (Li et al. 2019). Although HQD has been widely used for treating UC in China, its underlying mechanisms are still not clear.

This study investigates the effect of IFN-γ/JAK/ETS signalling on apoptosis of colonic epithelial cells in mice with UC induced by a hot and humid environment, a high-fat diet and a low concentration of dextran sodium sulphate. Moreover, we further explored the potential biological mechanism of HQD for the treatment of UC.

## Materials and methods

### Animals and medicines

Eight-week-old specific pathogen-free male C57BL/6 mice were purchased from Beijing HFK Bioscience Co., Ltd. (Beijing, China). All experiments were approved by the Animal Experiment Ethics Committee at Jinan University (ethics number: 2020521-12), and the animal study program was performed in accordance with the approved guidelines. The mice were transplanted in the Guangzhou Key Laboratory of Formula-Pattern of Traditional Chinese Medicine (Guangzhou, China). All mice were housed in cages under laboratory conditions with free access to food and water. The herb composition and specific information of HQD are shown in Table 1. All herbs were purchased from Guangdong Yifang Pharmaceutical Co., Ltd. (Guangzhou, China) and analysed with UPLC-Q/TOF-MS system. Dextran sodium sulfate (DSS) was purchased from MP Biomedicals (Irvine, CA) and dissolved in pure water at a concentration of 2.5% before use. Salazosulfapyridine (SASP) was purchased from Macklin (Shanghai, China). It was dissolved in pure water before use, and mice were treated with a dose of 500 mg/kg.

**Table 1.** Composition information of each Chinese herbal medicine in HQD.

Chinese pinyin name	Latin name	Composition ratio
Gan cao	*Glycyrrhiza uralensis* Fisch	6
Bai shao	*Paeonia lactiflora* Pall	6
Huang qin	*Scutellaria baicalensis* Georgi	9
Da zao	*Ziziphus jujube* Mill	49

### UPLC-Q/TOF-MS analysis

Preparation of samples: take 1 g of particles, add 10 mL of pure methanol to dissolve, sonicate for 10 min, centrifuge at 14,000 rpm for 10 min, and 2 μL of the supernatant is analysed by ultra-performance liquid chromatography to quadrupole time-of-flight mass spectrometry (UPLC-Q/TOF-MS).

UPLC-Q/TOF-MS analysis: UPLC analysis was performed on an Acquity UPLC 1-Class system (Waters Corporation, Milford, MA) equipped with a binary solvent system, autosampler. Chromatographic separation was achieved using a BEH C18 column (2.1 mm × 100 mm, 1.7 μm) at a column temperature of 40 °C. The mobile phase consisted of water (A) and acetonitrile (B), both containing 0.1% formic acid (v/v). Gradient elution was performed at a flow rate of 0.4 mL/min, and the elution program was as follows: 0–3 min, 5–11% B, 3–20 min, 11–40% B, 20–25 min, 40–60% B, 25–28 min, 60–100% B, 28–30 min, 100–100% B, 30–32 min, 100–5% B. The injection volume was set to 2 μL. The UPLC system was in tandem with a quadrupole time-of-flight mass spectrometer (SYNAPT G2 HDMS, Waters, Manchester, UK). Electrospray ionization (ESI) source was used for mass spectrometry, capillary voltage 3 kV (ESI+) or −2.5 kV (ESI–), cone voltage 30 V (ESI+) or 40 V (ESI–), secondary cone voltage 4 V, source temperature 100 °C, desolvation temperature 300 °C, backflush gas flow 50 L/h, desolvation gas flow 800 L/h, argon is the collision gas, in MSE mode, argon is used as the collision gas for CID. The mass spectrum was scanned in the range of 50–1500 Da, the mass axis was corrected with sodium formate solution, and the mass accuracy was corrected with leucine enkephalin as the internal standard (positive ion mode *m/z* 556.2771). Data collection is in centroid mode.

### Group and modelling method

After one week of adaptive feeding, mice were divided into four groups as follows: (i) normal control (NC, *n* = 10); (ii) model (UC, *n* = 10); (iii) model + SASP (UC + SASP, *n* = 10); and (iv) model + HQD (UC + HQD, *n* = 10). The NC group was kept in a normal environment (22 ± 2 °C, 60 ± 10% humidity, 12 h light/dark cycle) and had free access to food and pure water. To induce UC, three groups of mice, UC, UC + SASP and UC + HQD, were fed a high-fat and high-sugar diet (60% fat, 20% protein and 20% carbohydrate; purchased from the Center of Laboratory Animal Science of Guangdong, Guangdong, China). At the same time, the mice were housed in an artificial climate box (temperature 32 °C, humidity 90%, for 16 h a day). After 15 days of continuous administration to the model mice with damp heat factors and high fat and high sugar, the model mice were transferred to a normal breeding environment and given normal feed. Meanwhile, 2.5% DSS was prepared for free drinking by the model mice. After 1 week, the model mice were given free access to pure water, and each group was administered the corresponding drugs. The UC group was administered the corresponding pure water, the UC + SASP group was given SASP (500 mg/kg), and the UC + HQD group was given HQD (9.1 g/kg) once a day for seven consecutive days.

### Sample preparation

During the modelling process, the model was evaluated by observing the weight, diet, hair lustre, mental state, activity and stool characteristics of mice. After fasting for 12 h, all mice were anaesthetized with isoflurane (RWD Life Science Co., Ltd., Shenzhen, China). The colon was dissected out, washed with pre-cooled physiological saline, and then laid flat on filter paper to measure its length. Finally, the mid-end colon tissue was collected and stored at −80 °C for further analysis.

### HE staining and histopathological score

Approximately, 0.5 cm of the middle colon was placed on ice and fixed in 4% formaldehyde. After the tissue was dehydrated, it was made into paraffin sections. The sections were stained with haematoxylin and then dehydrated, immersed in eosin staining solution, dehydrated again, and then mounted with neutral gum. Tissue sections were evaluated, and images were obtained using a standard light microscopy on an Olympus microscope (Olympus Corporation, Tokyo, Japan). Tissue samples were well oriented with longitudinally cut crypts to precisely assess alterations in the overall intestinal tissue architecture. The scoring scheme of colonic histopathology is shown in Table 2 (Erben et al. 2014).

**Table 2.** The scoring scheme of colonic histopathology.

Inflammatory cell infiltrate		Score 1	Intestinal architecture		Score 2
Severity	Extent		Epithelial changes	Mucosal architecture	
Mild	Mucosa	1	Focal erosions		1
Moderate	Mucosa and submucosa	2	Erosions	± Focal ulcerations	2
Marked	Transmural	3		Extended ulcerations ± granulation tissue ± pseudopolyps	3
			Sum of scores 1 and 2		0–6

### Ultrastructure

Colon tissue (2 × 2 mm) was collected on ice, preserved and dehydrated. Tissues were embedded and polymerized by the permeation embedding method, and the tissue was made into 60–80 nm ultrathin sections. The sections were stained with 2% uranyl acetate saturated alcohol solution and 2.6% lead citrate solution and observed under a transmission electron microscope (Hitachi, Shiga, Japan).

### Detection of serum inflammatory factors

After anaesthesia, approximately 0.8 mL of blood was collected from the mouse orbit. After serum was collected by centrifugation, the levels of TGF-β1, TNF-α, IFN-γ, IL-4, IL-8, IL-10 and IL-17 in the serum were detected using enzyme-linked immunosorbent assay. The microplate was washed, serum of mice (20 μL per well) and standards were added, and samples were incubated with shaking at room temperature for 2 h. After washing six times, horseradish peroxidase-labelled streptavidin was added to the wells and incubated with shaking at room temperature for 45 min. After mixing, the samples were washed again six times, TMB, a chromogenic substrate, was added, and the mixture was incubated for 30 min in the dark at room temperature. Finally, the stop solution was added, and the OD value was measured using a microplate reader (BioTek, Winooski, VT) within 30 min.

### Western blot analysis

Total protein was extracted from colon tissue using RIPA Lysis buffer (Beyotime, Shanghai, China) supplemented with 1% protease inhibitor cocktail (Roche Diagnostics, Basel, Switzerland) and 1% phosphatase inhibitor (Millipore, Billerica, MA). The protein concentration in the lysate was determined using the Pierce BCA protein assay kit (Thermo, Waltham, MA). Based on this, a protein loading solution with a concentration of 3 μg/μL was prepared. After boiling the samples for 10 min, equal amounts of protein (15 μg) were separated by sodium dodecyl sulphate-polyacrylamide gel electrophoresis and electrophoretically transferred to polyvinylidenedifluoride membranes (Millipore, Billerica, MA). Blocking was performed by incubating the membranes for 1 h at room temperature in 5% non-fat, dry milk diluted in TBS containing 0.1% Tween-20. The membranes were then incubated overnight with primary antibodies against IFN-γ (1:1000, Cell Signaling Technology, Boston, MA), IFITM3 (1:1000, Cell Signaling Technology, Boston, MA), JAK1 (1:1000, Cell Signaling Technology, Boston, MA), JAK2 (1:1000, Cell Signaling Technology, Boston, MA), ETS-1 (1:1000, Cell Signaling Technology, Boston, MA), STAT1 (1:1000, Cell Signaling Technology, Boston, MA) and Bcl2 (1:1000, Cell Signaling Technology, Boston, MA) at 4 °C with gentle shaking. Membranes were then incubated with the secondary antibody for 1 h. Protein expression levels were analysed using ChemiDoc MP (Bio-Rad, Hercules, CA). β-Actin (Santa Cruz, Santa Cruz, CA) was used as an internal control.

### Statistical analysis

Data are presented as the mean ± standard error of the mean (SEM) and were statistically analysed with SPSS 20.0 (IBM Corp., Armonk, NY). In multiple group experiments with parametric data, the differences between groups were assessed using one-way ANOVA to determine overall significance followed by Dunnett’s multiple comparison test. The Kruskal–Wallis *H* test was used for ranked data. In the comparison of the body weight of each group, two-way ANOVA with repeated measures was used to determine significant differences.

## Results

### HQD alleviates the general symptoms of UC mice

**Figure 1.**
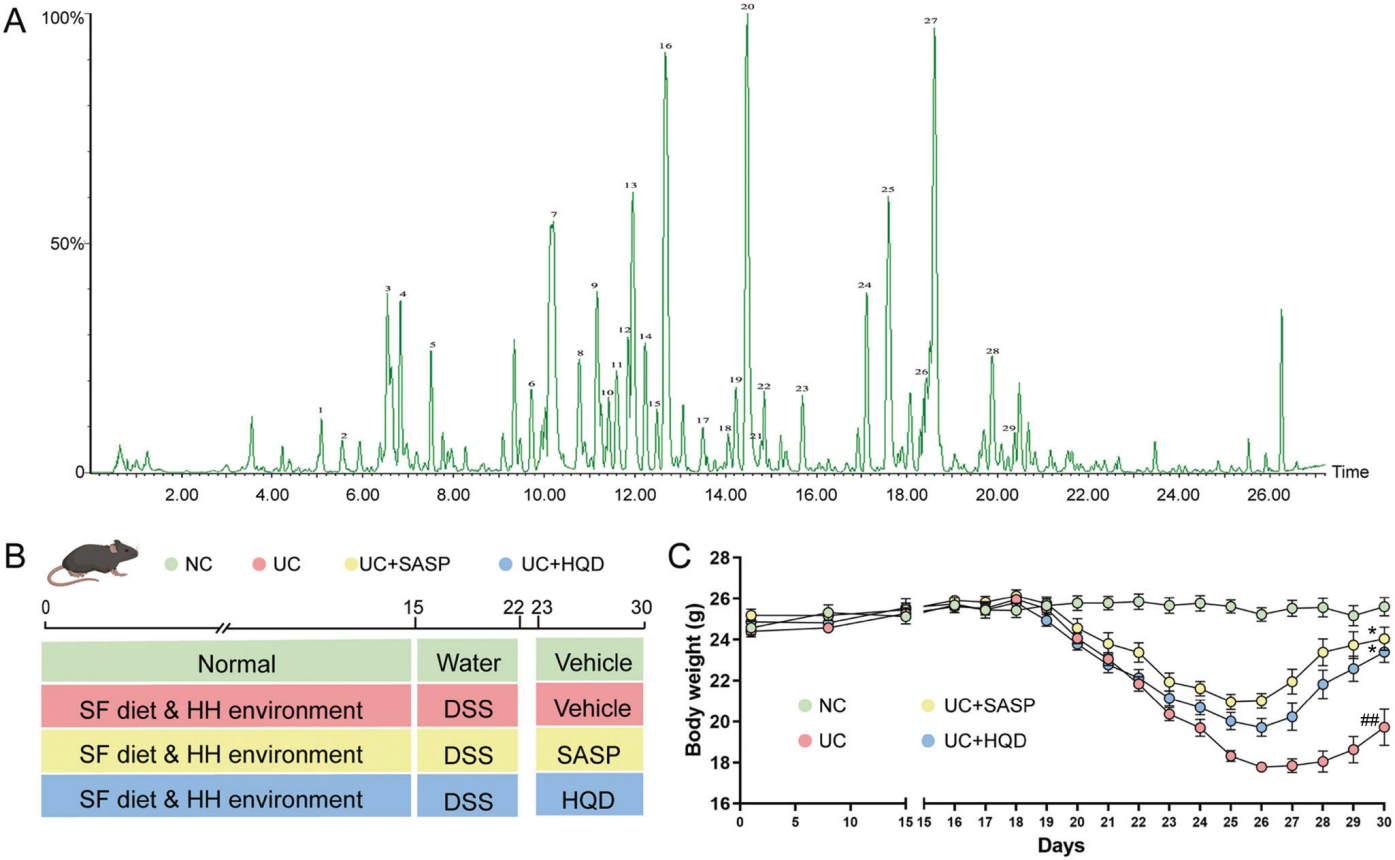
Preliminary characterization of HQD and experiment process. (A) Mass spectrum of the positive ion peak of HQD. The numbers in the figure indicate the identified compounds, and the specific information is shown in Table S1. (B) Schematic diagram of the specific experimental process. SF diet: high-sugar and high-fat diet; HH environment: hot and humid environment. (C) Body weight growth curve of the four groups. Two-way ANOVA with repeated measures showing significant differences. ^##^*p* < 0.01, vs. NC; ^*^p < 0.05, vs. UC.

In this study, we were concerned not only about colonic ulcer lesions directly caused by DSS but also about the influence of external environmental factors and dietary factors on UC. Therefore, we used a compound modelling method to establish a UC model and administered HQD as an intervention drug to examine the biological mechanism of HQD in the treatment of UC. To ensure the rigour of the experiment and the reproducibility of subsequent experiments, we used UPLC-Q/TOF-MS to initially characterize the composition of HQD (Figure 1(A), Table S1). The specific experimental process is shown in Figure 1(B). Model mice in this study exhibited severe UC-related symptoms, such as diarrhoea, stiff hair and bloody stools. The body weight change graph (Figure 1(C)) illustrates that previous exposure to the humid and hot environment and high-fat and high-sugar factors did not significantly affect the weight of mice. After using DSS in the later stage, the weights of mice in the UC group, UC + SASP group and UC + HQD group decreased rapidly compared to the NC group; when treated with SASP or HQD, the weights of the mice gradually recovered.

### HQD attenuates colon damage in ulcerative colitis

**Figure 2.**
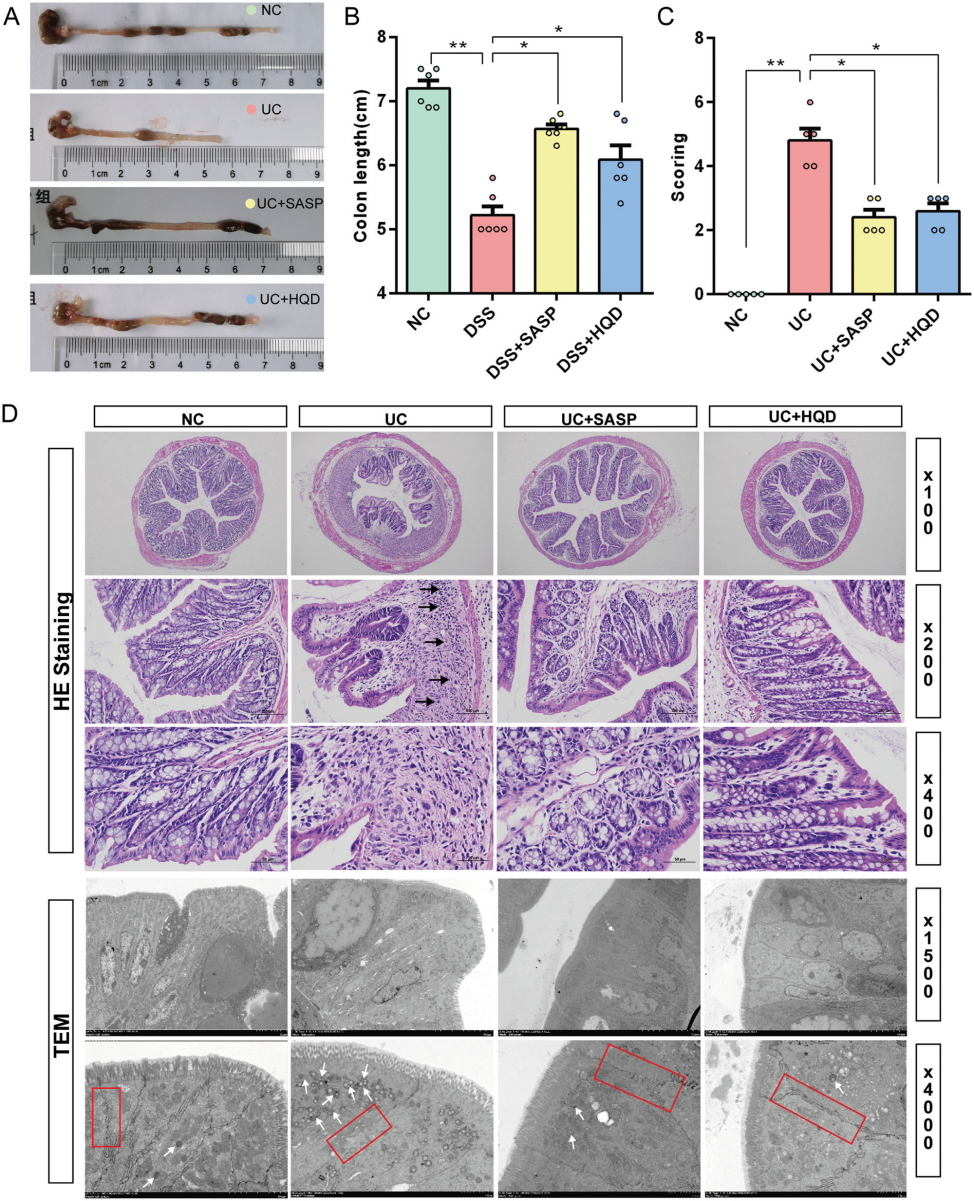
HQD attenuates colon damage in ulcerative colitis. (A) Representative images of colon tissue in each group. (B) Histogram of colon length (*n* = 6). Dunnett’s test showed significant differences. (C) Histogram of colonic pathology score (*n* = 5). The Kruskal–Wallis *H* test showing significant differences. (D) Representative images of haematoxylin–eosin staining (HE staining) and transmission electron microscopy (TEM). The black arrow shows the submucosal infiltration of inflammatory cells. The white arrow shows a lysosome. The red box shows an apical tight junction. Data are presented as the mean ± SEM for each group of mice (*n* = 6); *p < 0.05, **p < 0.01.

Observing the length of the mouse colon with the naked eye (Figure 2(A,B)), colons from the NC group were approximately 38.02% longer than those in the UC group (*p* < 0.01), and when treated with HQD or SASP, the colon length of mice in the UC + HQD group and UC + SASP group recovered significantly, being approximately 16.61% (*p* < 0.01) and 25.88% (*p* < 0.01) longer than the UC group.

Observing HE-stained sections of the colon, it was found that the colon gland structure of the NC group was intact and undamaged, there was no ulcer formation in the submucosal tissue, and there was no obvious inflammatory cell infiltration. In the UC group, the colon exhibited obvious erosion or ulcer formation, the crypt structure under the mucosa was deformed, and the inflammatory cells at the base of the mucosa were increased (shown by the black arrow in Figure 2(D)). After SASP or HQD treatment, colon tissue morphology was significantly improved, as shown in Figure 2(D). We next scored the degree of colonic lesions. Compared to the NC group, the colon score of mice in the NC group was significantly increased (*p* < 0.01), while the lesion scores of the UC + SASP group and UC + HQD group were lower than those of the UC group, as shown in Figure 2(C). To further observe the changes in the microstructure of colonic epithelial cells, we performed transmission electron microscopy. In the UC group, the colonic epithelial columnar cells exhibited sparse microvilli on the surface, intracellular mitochondrial enlargement, and mitochondrial ridges, while other organelle structures were blurred with the number of intracellular lysosomes being significantly increased (shown by the white arrow in Figure 2(D)). After HQD treatment, the microvilli on the surface of colonic epithelial cells in the UC + HQD group increased, the mitochondria were not enlarged, the mitochondrial ridges and other organelle structures were clearly visible, the number of intracellular lysosomes was moderate, and the cell microstructure was improved. The apical tight junction (TJ) is an important intercellular structure between colonic epithelial cells (shown in the red box in Figure 2(D)). This structure forms the intestinal mucosal barrier and protects the submucosal tissues from bacterial products in the intestinal lumen (Bruewer et al. 2006). The TJ structure between colonic epithelial cells in the UC group was damaged, and the intercellular space increased, while the TJ structure in the UC + HQD group was significantly improved, and the intercellular space returned to normal compared to the UC group.

### HQD modulates cytokine expression to regulate serum inflammation in ulcerative colitis

**Figure 3.**
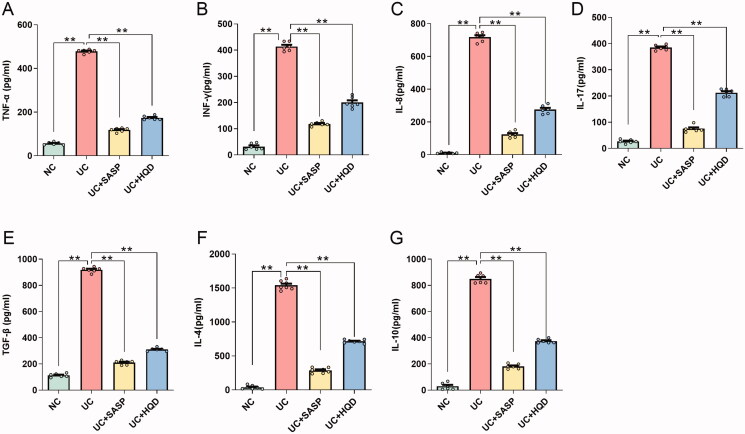
The effect of HQD on serum inflammatory factors in mice with ulcerative colitis. (A–G) Histograms of TNF-α, IFN-γ, IL-8, IL-17, TGF-β, IL-4 and IL-10 in the serum of each group of mice. TNF-α: tumour necrosis factor α; IFN-γ: interferon gamma; IL-8: interleukin 8; IL-17: interleukin 17; TGF-β: transforming growth factor beta; IL-4: interleukin 4; IL-10: interleukin 10. Data are presented as the mean ± SEM. for each group of mice (*n* = 6); **p < 0.01.

In the pathogenesis of UC, there are not only abnormalities in the local immune function of the intestinal mucosa but also systemic immune disorders. Cytokines are involved in the immune response and inflammatory process. To evaluate the overall proinflammatory and anti-inflammatory systems in mice, we assessed levels of proinflammatory cytokines (IL-8, TNF-α, IL-17 and IFN-γ) and anti-inflammatory cytokines (TGF-β, IL-4 and IL-10) in the serum (Figure 3). The results showed that whether pro-inflammatory cytokines or anti-inflammatory cytokines were present, the levels of these cytokines in the UC group were significantly higher than those in the NC group. This result suggests that the host may trigger proinflammatory and anti-inflammatory systems during the pathogenesis of acute UC, resulting in increased inflammatory cytokines. After treatment, serum levels of IL-8, TNF-α, IL-17, IFN-γ, TGF-β, IL-4 and IL-10 in the UC + HQD group were significantly lower than those in the UC group, indicating that HQD improved the immune function of the host system, regulated the proinflammatory and anti-inflammatory systems, and reduced inflammatory damage in model mice.

### HQD reduces colonic epithelial cell apoptosis in ulcerative colitis

**Figure 4.**
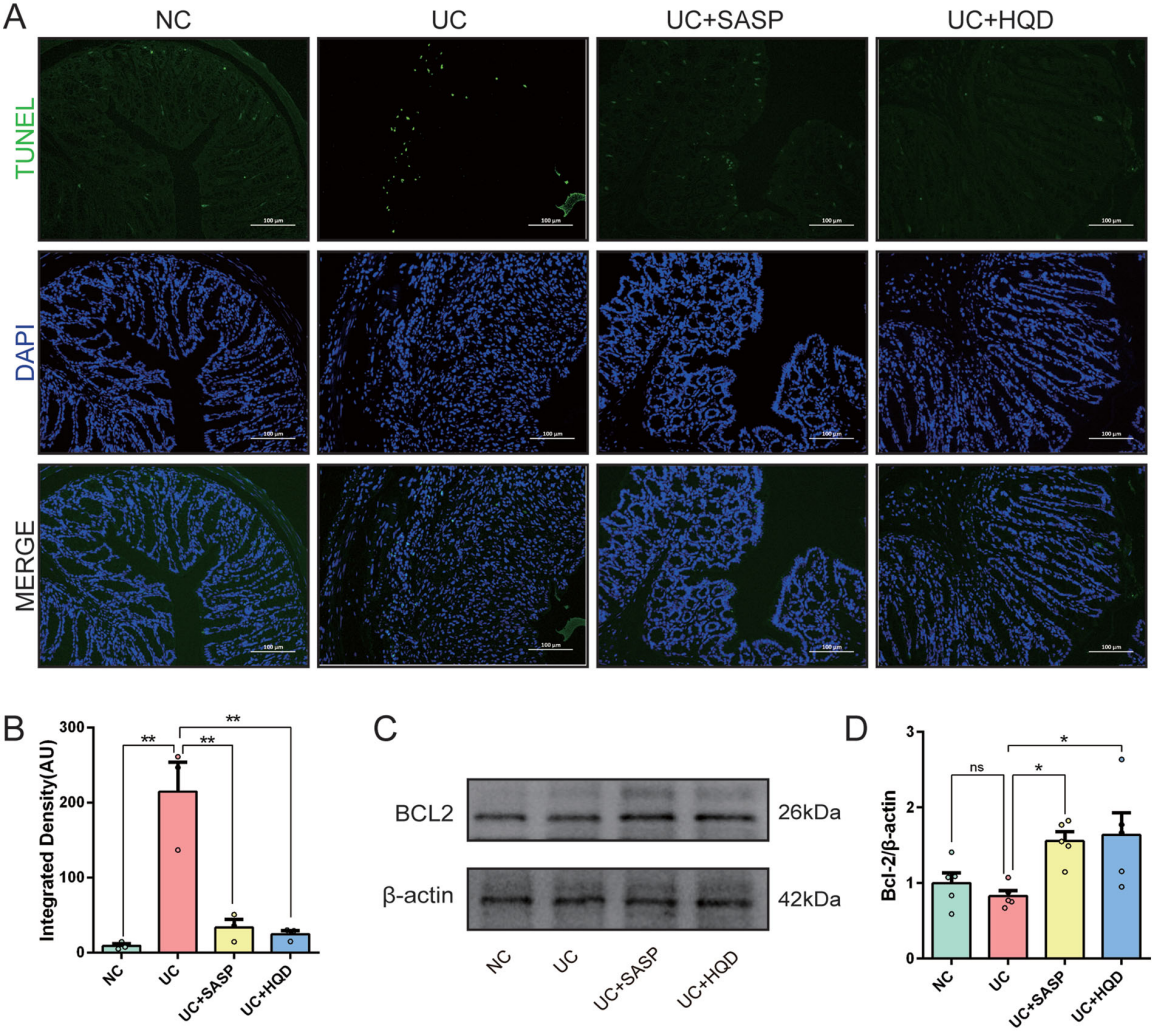
HQD reduces colonic epithelial cell apoptosis in ulcerative colitis. (A) TUNEL staining of colonic tissues from the treated mice (original magnification, ×200). (B) Fluorescence intensity of each group for TUNEL staining (*n* = 3). The Kruskal–Wallis *H* test showed significant differences. (C) Representative western blot and semiquantitative analysis of BCL-2 protein in each group. (D) Histogram of BCL-2 protein content in colon tissue (*n* = 5). Data are presented as the mean ± SEM for each group of mice; *p < 0.05, **p < 0.01.

Studies have shown that excessive apoptosis is one of the important mechanisms of the pathogenesis of UC. Proinflammatory cytokines produced in response to colonic mucosal injury and immune abnormalities can excessively increase cell apoptosis and aggravate the development of UC (Iwamoto et al. 1996; Zeng et al. 2015). TUNEL staining (Figure 4(A,B)) showed that there were excessive apoptotic cells in the colon of the UC group, and their fluorescence intensity was significantly higher than that of the NC group (*p* < 0.01). After HQD treatment, the number of apoptotic cells in the colon in this group of mice was reduced compared to the UC group, and their fluorescence intensity was significantly lower than that of the UC group (*p* < 0.01). At the same time, we detected a key protein indicator, B-cell lymphoma-2 (Bcl-2), in apoptosis, as shown in Figure 4(C). The protein expression of Bcl-2, which exerts an antiapoptotic effect, was reduced in the UC + HQD group (*p* < 0.05). The above results indicate that HQD reduces apoptosis in colon cells and slows the development of UC.

### HQD attenuates ulcerative colitis through the IFN-γ/JAK/ETS signalling pathway

**Figure 5.**
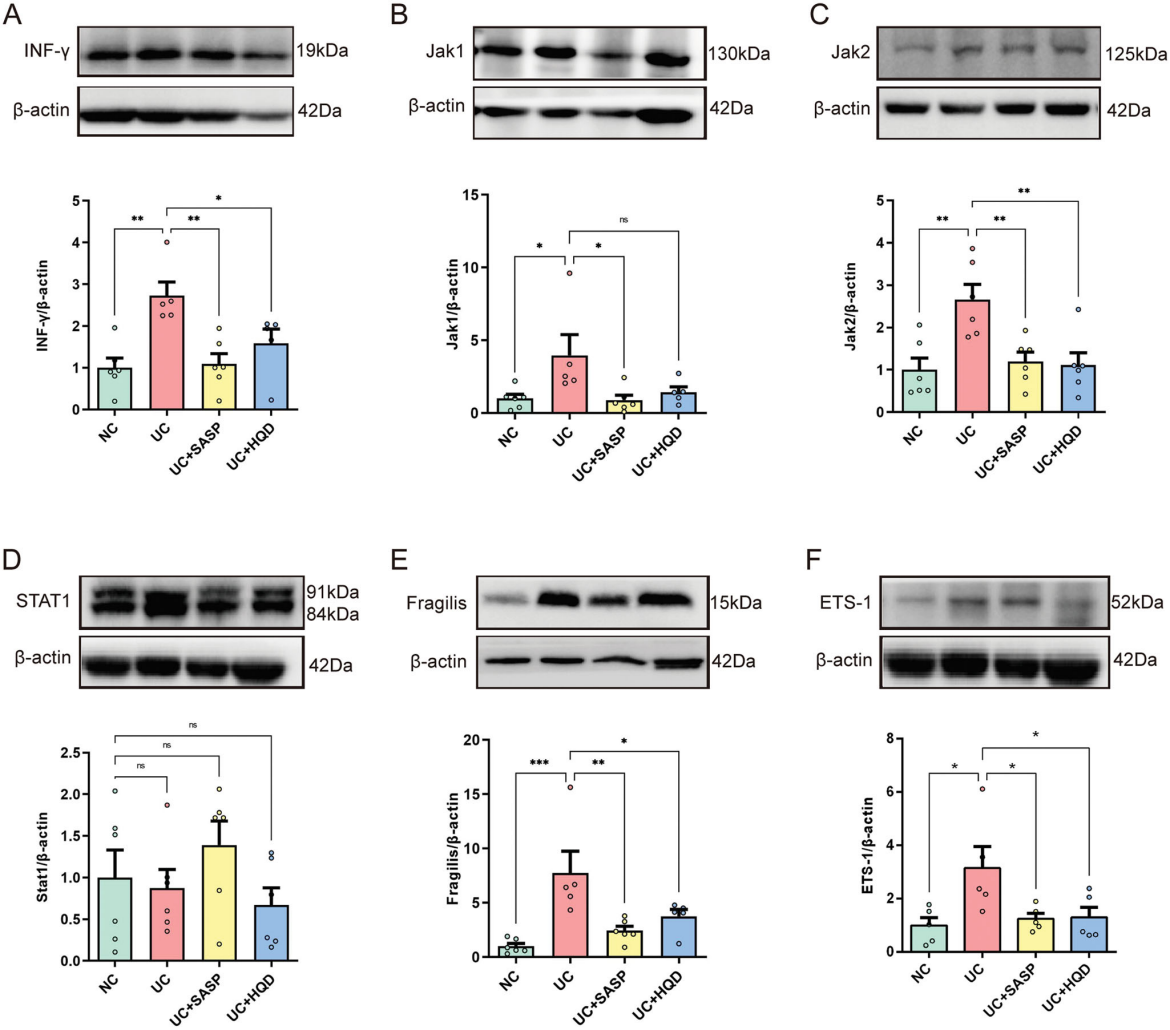
Western blot analysis of protein levels associated with the IFN-γ/JAK/ETS pathway in colon tissue. (A) Representative western blot and semiquantitative analysis of IFN-γ protein in each group. (B) Representative western blot and semiquantitative analysis of JAK1 protein in each group. (C) Representative western blot and semiquantitative analysis of JAK2 protein in each group. (D) Representative western blot and semiquantitative analysis of Stat1 protein in each group. (E) Representative western blot and semiquantitative analysis of fragilis protein in each group. (F) Representative western blot and semiquantitative analysis of EST-1 protein in each group. One-way ANOVA by Dunnett’s test was used for statistical analysis. Data are presented as the mean ± SEM for each group of mice (*n* = 5–6); *p < 0.05, **p < 0.01.

Next, to clarify the specific biological mechanism of excessive colon cell apoptosis, we started with immune abnormalities and conducted research on the IFN-γ/JAK/ETS signalling pathway, as shown in Figure 5. IFN-γ is a pleiotropic cytokine that has a more prominent immunomodulatory effect than its antiviral activity (Barrat et al. 2019). In this study, compared to the NC group, IFN-γ protein expression in the colon tissue of the UC group was significantly upregulated (*p* < 0.01). After HQD treatment, IFN-γ protein expression was significantly downregulated compared to the UC group (*p* < 0.05, Figure 5(A)). IFN-γ binds to type II interferon receptors on the cell surface to activate the downstream JAK/STAT signalling pathway, which is widely involved in cell proliferation, differentiation, apoptosis and inflammation (Darnell et al. 1994; Hu and Ivashkiv 2009). It can be seen from the figure that expression levels of JAK1, JAK2 and fragilis protein in the colon tissue of the UC group were significantly higher than those of the NC group (*p* < 0.05), indicating that the JAK/STAT signalling pathway was activated. In response to HQD treatment, protein expression levels of JAK2 (*p* < 0.01) and fragilis (*p* < 0.05) in the colon tissue of the UC + HQD group were downregulated to varying degrees (Figure 5(B–E)). The E26 transformation specific-1 (ETS) family of transcription factors is one of the largest families of transcriptional regulatory factors in cells and is regulated by the JAK/STAT signalling pathway and participates in the process of apoptosis. Consistent with the TUNEL results above, protein expression of ETS-1 in the colon tissue of the UC group was significantly upregulated compared to the NC group (*p* < 0.05), while HQD downregulated expression of ETS (*p* < 0.05, Figure 5(F)). The above results indicate that the IFN-γ/JAK/ETS signalling pathway in the colon tissue of the UC group is activated during the onset of disease, leading to excessive colon cell apoptosis. This study also implies that the IFN-γ/JAK/ETS signalling pathway may play an important role in the pathogenesis of UC, and HQD attenuates UC by regulating the IFN-γ/JAK/ETS signalling pathway.

## Discussion

The pathogenesis of UC is related to the complex interaction between genetic susceptibility and environmental factors, such as smoking, infection, sleep, climate change and diet (Du and Ha 2020). Among them, the role of climatic factors and dietary factors in the pathogenesis of UC cannot be underestimated. Studies have shown that changes in the outside climate or seasons have a certain impact on the process of UC, and the primary two variables of climate change (temperature and humidity) can affect the host’s immune function and species richness of the gut microbiome (Aamodt et al. 2013). At the same time, the common ‘Western-style’ diet (processed meats, refined carbohydrates, etc.) is associated with an increased risk of developing IBD. The increased intake of total fat, animal fat and polyunsaturated fatty acids in the diet increases the host's UC risk (Hou et al. 2011; Singh et al. 2017). To better reflect the complex pathogenesis of clinical UC patients, this study used a combination of factors, such as hot and humid climate, high-fat and high-sugar diet, and low-dose DSS, to establish a mouse UC model. The results of this study indicate that this compound modelling method successfully establishes a stable mouse model of UC. We believe this modelling method that integrates multiple factors may better reflect the clinical situation of UC patients.

Sulfasalazine is the first aminosalicylate used to induce UC remission and as a maintenance therapy. When sulfasalazine is used for a long time, there may be adverse events, such as liver and nephrotoxicity, drug resistance and allergic reactions, and these adverse events are attributed to the sulfapyridine moiety (Nikfar et al. 2009). HQD is a well-known traditional Chinese medicine formulation with a history of clinical application for approximately 2000 years. Clinical studies have shown that HQD significantly improves the clinical symptoms of UC patients and reduces the recurrence rate (Li et al. 2019). In this study, we used HQD as an intervention drug to study the biological mechanism of HQD in the treatment of UC. The results showed that after HQD treatment, the weight of UC model mice gradually increased, the length of the colon was significantly restored, the pathological damage of the colon tissue in model mice was attenuated, and the infiltration of inflammatory cells into colon tissue was reduced. Transmission electron microscopy revealed that the TJs between the colonic epithelial cells of the UC group were significantly abnormal compared to the NC group, and the TJs between the cells were reduced. This barrier controls the transport of molecules through transcellular and paracellular pathways, and a dysfunctional intestinal TJ barrier increases the penetration of antigens, endotoxins and bacteria into the bloodstream in the cavity (Dokladny et al. 2016). Studies have shown that destruction of the epithelial barrier is the basis of IBD and is related to the severity of UC (Parikh et al. 2019). HQD not only reduced damage to the intestinal mucosa but also improved the abnormal structure of TJs in terms of ultrastructure and restored the function of the barrier intestinal mucosa.

UC is not simply an inflammatory ulcer in the colon tissue but also involves systemic immune dysfunction in the host, as well as an imbalance in the proinflammatory and anti-inflammatory systems (Gajendran et al. 2019). During the pathogenesis of UC, the interaction between proinflammatory and anti-inflammatory factors has a profound impact on the development direction and outcome of UC (Tatiya-Aphiradee et al. 2018). Increases in the levels of proinflammatory cytokines, such as IFN-γ, TNF-α, IL-8 and IL-17, are observed with immune enhancement (activation of T and B cells, synergistic effect with other cytokines and promotion of cytokine gene expression) and inflammatory effects (induction of fever, lead to arachidonic acid metabolism and increase in collagenase synthesis). In contrast, anti-inflammatory cytokines, such as TGF-β, IL-4 and IL-10, suppress immunity, reduce the activation of lymphocytes, and inhibit the aggregation of neutrophils (Park et al. 2017). A prospective clinical study on UC identified a correlation between the activity of UC and the levels of serum IL-13 and IL-17, among which the serum level of IL-17 is significantly correlated with disease severity (Boldeanu et al. 2014). Studies have shown that host IL-10 polymorphisms may affect susceptibility to IBD (Su and Zhao 2020). In this study, we examined proinflammatory and anti-inflammatory cytokines in the serum of mice and found that the expression levels of IFN-γ, TNF-α, IL-8, IL-17, TGF-β, IL-4 and IL-10 in the UC group were significantly higher than those in the NC group. This indicates that the host's proinflammatory and anti-inflammatory systems are both activated in acute colitis injury. HQD significantly reduced proinflammatory and anti-inflammatory cytokines, indicating that HQD attenuates intestinal inflammatory damage by regulating the host's proinflammatory and anti-inflammatory systems.

Excessive apoptosis of colon tissue cells damages the integrity of the epithelial barrier and promotes the pathogenesis of UC. We evaluated the apoptosis of the colon tissue of each group of mice. As previously assumed, there was excessive cell apoptosis in the colon of mice in the UC group (Wu et al. 2017), and HQD alleviated the apoptosis of colon cells in model mice. In this study, we found that expression of IFN-γ in the serum and colon tissue of the UC group was significantly increased and that the expression levels of IFN-γ were significantly downregulated after HQD treatment. IFN-γ, a pleiotropic anti-inflammatory cytokine, is a key factor in driving cellular immunity and coordinates a variety of protective functions to enhance the immune response in infection and cancer. It exerts its immunoregulatory effects by enhancing antigen processing and presentation, increasing leukocyte trafficking, inducing antiviral status, enhancing antimicrobial function and affecting cell proliferation and apoptosis (Kak et al. 2018). The JAK/STAT signalling pathway is a classic downstream signalling pathway by which IFN-γ induces apoptosis, and it is involved in the pathogenesis of IBD (Horvath 2004; Coskun et al. 2013). After IFN-γ binds to the IFN receptor, it increases JAK kinase activity, and JAK itself is phosphorylated, providing ‘docking sites’ for STAT1 and STAT2 and leading to phosphorylation of STAT protein. Through the complex downstream signalling pathway, the JAK/STAT signalling pathway acts on the nucleus to promote transcription of a variety of interferon-stimulated genes.

In this study, we found that the protein expression of related proteins in the JAK/STAT signalling pathway in the colon tissue of the UC group, such as JAN1, JAN2 and fragilis, was significantly upregulated compared to the NC group, indicating that the JAK/STAT signalling pathway in the UC group was activated. EST-1 is the prototypic member of a novel subset of the ETS transcription factor family, and its expression in the gastrointestinal tract is particularly high. The JAK/STAT signalling pathway regulates the functional activity of EST family proteins and participates in the process of cell apoptosis (Oikawa and Yamada 2003). Recently, experimental studies have shown that EST-1 promotes the progression of UC by accelerating the activation of NF-κB to promote intestinal epithelial apoptosis (Li et al. 2015).

## Conclusions

We believe that the involvement of the IFN-γ/JAK/ETS signalling pathway in the process of regulating intestinal cell apoptosis is an important biological mechanism for the occurrence and development of UC. HQD reduced colon cell apoptosis and related inflammatory diseases by regulating the IFN-γ/JAK/ETS signalling pathway to attenuate UC.

## Author contributions

J.W. conceived and designed the experiments; X.M. and K.T. performed the experiments; L.D. and X.Z. analysed the data; X.L. and Y.Z. contributed reagents/materials/analysis tools; and K.T. wrote the manuscript. All authors agree to be accountable for the content of the work.

## Supplementary Material

Supplemental Material
