# Supplementary material for: Prevention of ulcerative colitis by Huangqin decoction: reducing the intestinal epithelial cell apoptosis rate through the IFN-γ/JAK/ETS signalling pathway
Source: Pharm Biol. 2022 Jun 2;60(1):1116–25. doi: 10.1080/13880209.2022.2070220 (PMC9176677; doi:10.1080/13880209.2022.2070220)
Supplement: Supplemental Material [file IPHB_A_2070220_SM8791.docx]

# Supplementary Table

Table S1: Specific information of HQD-derived compounds that have been marked in the positive ion peaks.

| NO. | Elemental composition | Identification | From |
| --- | --- | --- | --- |
| 1 | C24H29O13 | Albiflorin | BS |
| 2 | C24H29O13 | Paeoniflorin | BS |
| 3 | C21H22O9 | liquiritin | GC |
| 4 | C26H28O13 | Xylosylpuerarin | DZ |
| 5 | C26H28O13 | Mirificin | DZ |
| 6 | C21H22O9 | Isoliquiritin | GC |
| 7 | C21H18O11 | Baicalin | HQ |
| 8 | C21H20O11 | Dihydrobaicalin | HQ |
| 9 | C21H18O11 | Baicalein‐O‐glucuronide | HQ |
| 10 | C21H18O11 | Apigenin‐O‐glucuronoside | HQ |
| 11 | C22H20O12 | Scutellarein‐methyl ether‐O-glucuronide | HQ |
| 12 | C21H18O10 | Chrysin‐O‐glucuronide | HQ |
| 13 | C22H20O11 | Oroxylin A‐O‐glucuronide | HQ |
| 14 | C22H20O12 | Chrysoeriol‐O‐glucuronide | HQ |
| 15 | C21H18O11 | Norwogonin‐O‐glucuronide | HQ |
| 16 | C22H20O11 | Wogonoside | HQ |
| 17 | C15H20O5 | Norwogonin | HQ |
| 18 | C42H63O18 | 22-Hydroxy-licorice-saponin G2 | GC |
| 19 | C16H12O6 | Tenaxin II | HQ |
| 20 | C15H20O5 | Baicalein | HQ |
| 21 | C16H12O6 | Scutevulin | HQ |
| 22 | C48H73O21 | Licoricesaponin A3 | GC |
| 23 | C42H62O17 | Licorice-saponin G2 OR AMacedonoside A OR 22-Hydroxyl-glycyrrhizin OR Isomers | GC |
| 24 | C42H62O17 | Licorice-saponin G2 OR AMacedonoside A OR 22-Hydroxyl-glycyrrhizin OR Isomers | GC |
| 25 | C42H62O17 | Licorice-saponin G2 OR AMacedonoside A OR 22-Hydroxyl-glycyrrhizin OR Isomers | GC |
| 26 | C42H62O17 | Licorice-saponin G2 OR AMacedonoside A OR 22-Hydroxyl-glycyrrhizin OR Isomers | GC |
| 27 | C42H62O16 | Uralsaponin B OR Licorice-saponin H2 OR Glycyrrhizic acid OR Isomers | GC |
| 28 | C42H62O16 | Uralsaponin B OR Licorice-saponin H2 OR Glycyrrhizic acid OR Isomers | GC |
| 29 | C42H62O16 | Uralsaponin B OR Licorice-saponin H2 OR Glycyrrhizic acid OR Isomers | GC |

Note. BS, Bai Shao; GC, Gan Cao; DZ, Da Zao; HQ, Huang Qin.
